# Cross-sectional description of early postnatal fatty acid patterns in delivered preterm infants

**DOI:** 10.1016/j.isci.2026.116882

**Published:** 2026-07-24

**Authors:** Chuan Zhang, Zhao Wang, Hui Wu, Lin Xie, Haitao Yu, Dan Dang

**Affiliations:** 1 Department of Pediatric Surgery, Children’s Medical Center, First Hospital of Jilin University, Changchun 130021, China; 2 Department of Laboratory Medicine, First Hospital of Jilin University, Changchun, China; 3 Department of Neonatology, Children’s Medical Center, First Hospital of Jilin University, Changchun 130021, China; 4 Department of Nutrition and Food Hygiene, School of Public Health, Jilin University, Changchun 130021, China

**Keywords:** preterm infants, fatty acid profile, gestational age, perinatal factors

## Abstract

Preterm birth prematurely interrupts maternal fatty acid supply, yet gestational age-specific serum fatty acid profiles and perinatal determinants remain incompletely defined. In this prospective cohort, serum samples of very preterm infants (<32 weeks) collected within 24 h of birth were analyzed by gas chromatography-mass spectrometry. Fatty acids were classified as SCFAs, MCFAs, LCFAs, MUFAs, and PUFAs. Associations with gestational age, maternal comorbidities, antenatal medications, and infant sex were assessed using multivariable linear regression. A total of 105 very preterm infants were included. Lower gestational age was associated with greater fatty acid insufficiency. Total SCFAs increased with gestational age (*p* = 0.003), driven by rising butyric acid (C4:0) and caproic acid (C6:0). SFAs and MUFAs increased significantly (*p* = 0.005 and 0.04) with gestational age. Although total LCFAs and PUFAs did not differ significantly, the proportion of linoleic acid increased with gestational age (C18:2n6c) (*p* = 0.01). Maternal hypertension was associated with higher proportions of C18:2n6c (*p* = 0.001) and decreased levels of eicosatrienoic acid (C20:3n6, *p* = 0.04) and arachidonic acid (ARA, C20:4n6; *p* = 0.05). Infants of diabetic mothers showed an elevated ARA/DHA (docosahexaenoic acid, C22:6n3) ratio (*p* = 0.003). In multiple gestation, the C18:2n6c proportion increased (*p* = 0.0007), and the DHA proportion decreased (*p* = 0.005). Male infants had lower levels and proportions of C20:3n6 than females (*p* = 0.009 and 0.003). Antenatal corticosteroids were associated with reduced levels and proportions of C6:0 (both *p* = 0.02), decreased C18:2n6c proportion (*p* = 0.003), and higher levels of ARA (*p* = 0.03) and DHA (*p* = 0.05). Antenatal magnesium sulfate exposure was linked to decreased levels and proportions of C16:1n7 (*p* = 0.04 and 0.05). Serum fatty acid profiles in preterm infants exhibit gestational age-dependent patterns and are associated with maternal comorbidities, antenatal medication exposure, and infant sex, providing new insights into early postnatal fatty acid status in this population.

## Highlights

• Fatty acid profiles differ significantly across gestational ages in very preterm infants
• Extremely preterm infants exhibit lower levels of SCFAs, SFAs, and MUFAs
• Maternal and perinatal factors are associated with specific fatty acid alterations

## Graphical abstract

**Figure:**
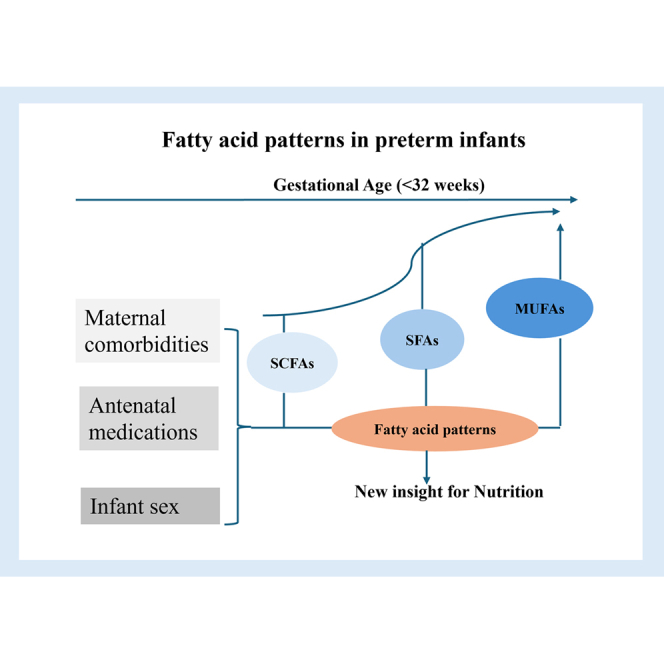

## Teaser

Clinical finding; Molecular biology

## Introduction

Sustaining extrauterine growth in preterm infants requires adequate nutritional support. Fatty acids function as both primary energy sources and structural elements of cellular membranes, while also regulating signaling, inflammation, and neurodevelopment.^1^^,^^2^^,^^3^^,^^4^ The premature interruption of placental fatty acid transfer in late gestation predisposes infants to deficiencies and metabolic dysregulation, which are considered potential contributors to impaired postnatal growth and an increased risk of morbidities such as bronchopulmonary dysplasia (BPD), retinopathy of prematurity (ROP), and adverse neurodevelopmental outcomes.^5^^,^^6^^,^^7^^,^^8^

Although neonatal fatty acid metabolism is a research hotspot, current studies mainly focus on exogenous fatty acid interventions or the association between fatty acid levels and adverse disease outcomes. Baseline fatty acid profiles in neonates, particularly in preterm infants, remain poorly characterized. Only a limited number of studies have examined neonatal fatty acid composition: one compared fatty acid levels across gestational ages in term infants,^9^ while another analyzed erythrocyte membrane fatty acids in 30 preterm infants but only relative to 10 term controls,^10^ without systematic stratification across different preterm gestational age groups. Gestational age-specific analyses are therefore scarce. Moreover, most interventional studies have focused on individual fatty acids such as arachidonic acid (ARA, C20:4n6) and docosahexaenoic acid (DHA, C22:6n3), neglecting the overall balance among saturated, monounsaturated, and polyunsaturated fatty acids.^11^^,^^12^^,^^13^^,^^14^ In addition, the potential impact of maternal complications, perinatal medication exposure, and nutritional factors—conditions frequently encountered in preterm populations—has not been systematically assessed in relation to neonatal fatty acid metabolism.

Comprehensive profiling of preterm-specific fatty acid metabolism, together with the assessment of perinatal influences, is therefore essential to delineate distinctive metabolic signatures and to inform individualized nutritional strategies aimed at improving long-term outcomes.

## Results

### Baseline characteristics of the study population

**Figure 1:**
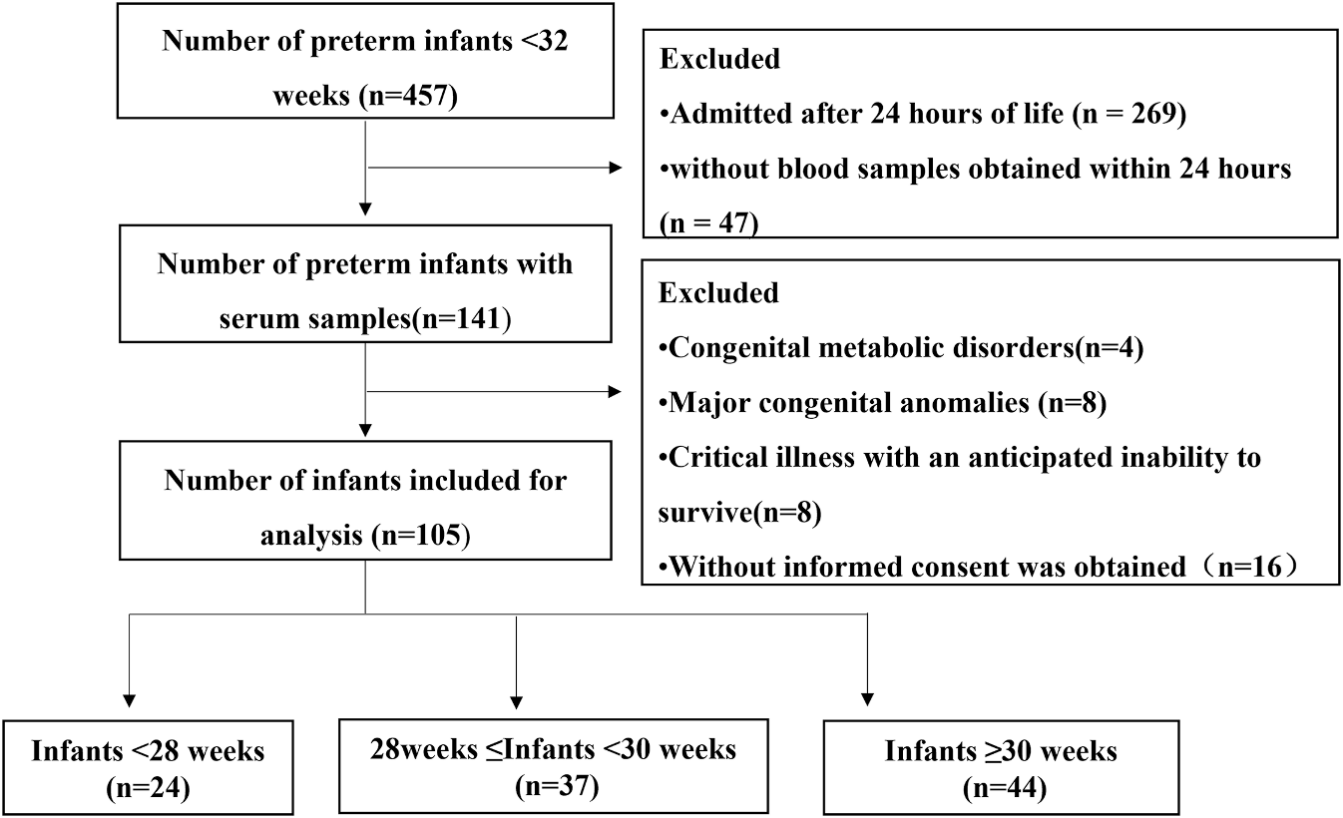
Flow chart of study population.

A total of 105 very preterm infants with a gestational age of <32 weeks were enrolled in this study (Figure 1), comprising 24 infants in the <28 weeks group, 37 in the 28–30 weeks group, and 44 in the ≥30 weeks group. The overall mean gestational age was 29.2 ± 1.8 weeks, and the mean birth weight was 1.2 ± 0.3 kg. No significant difference was observed in sex distribution among the three groups.

The incidences of respiratory distress syndrome (RDS, 89.5%), BPD (53.3%), and ROP (35.2%) increased significantly with decreasing gestational age, whereas the rates of NEC, PDA, IVH, and PVL did not differ significantly among groups. Mortality occurred exclusively in the <28 weeks group. The length of hospital stay decreased significantly with increasing gestational age. Maternal complications during pregnancy, including diabetes and hypertension, showed no significant differences among groups (Table 1).

**Table 1:** Baseline characteristics of the study population

Characteristics	Total (*n* = 105)	Gestational age <28 weeks (*n* = 24)	Gestational age 28–30 weeks (*n* = 37)	Gestational age 30–32 (*n* = 44)
Male sex (n, %)	55 (52.4)	14 (58.3)	19 (51.4)	22 (50.0)
Gestational age (week) (mean ± SD)	29.2 (1.8)	26.7 (1.2)	28.7 (0.5)	30.8 (0.7)
Birth weight (kg) (mean ± SD)	1.2 (0.3)	0.9 (0.2)	1.2 (0.3)	1.5 (0.3)
Maternal age (years) (mean ± SD)	32.7 (4.0)	34.0 (4.3)	32.7 (3.9)	32.0 (3.8)
IDM (n, %)	32 (30.5)	7 (29.2)	12 (32.4)	13 (29.5)
HIP (n, %)	23 (21.9)	3 (12.5)	11 (29.7)	9 (20.5)
C-section (n, %)	54 (51.4)	3 (12.5)	21 (56.8)	30 (68.2)
Multiple birth (n, %)	29 (27.6)	5 (20.8)	6 (16.2)	18 (40.9)
AC (n, %)	17 (16.2)	21 (87.5)	30 (81.1)	37 (84.1)
AS (n, %)	40 (38.1)	14 (58.3)	27 (73.0)	24 (54.5)
Apgar score@ 5 min < 5 (mean ± SD)	6 (5.7)	3 (12.5)	1 (2.7)	2 (4.5)
PDA (n, %)	33 (31.4)	10 (41.7)	11 (29.7)	12 (27.3)
ROP (n, %)	37 (35.2)	19 (79.2)	17 (45.9)	1 (2.3)
RDS (n, %)	94 (89.5)	24 (100.0)	36 (97.3)	34 (77.3)
BPD (n, %)	56 (53.3)	18 (75.0)	17 (45.9)	14 (31.8)
IVH (n, %)	39 (37.1)	12 (50.0)	10 (27.0)	17 (38.6)
NEC (n, %)	3 (2.9)	0 (0.0)	1 (2.7)	2 (4.5)
PVL (n, %)	1 (1.0)	1 (4.2)	0 (0.0)	0 (0.0)
Death (n, %)	2 (1.9)	2 (8.3)	0 (0.0)	0 (0.0)
Hospitalization days (day) (mean ± SD)	52.6 (23.0)	72.7 (29.1)	56.4 (11.8)	38.4 (16.2)
WBC (×10^9^/L) (mean ± SD)	10.9 (8.8)	14.2 (12.1)	10.4 (7.4)	9.7 (7.3)
Hb (g/L) (mean ± SD)	153.8 (23.7)	140.7 (21.0)	160.2 (24.3)	155.6 (22.3)
PLT (×10^9^/L) (mean ± SD)	227.3 (61.6)	249.6 (75.1)	222.7 (61.6)	219.1 (51.2)

IDM, maternal diabetes; HIP, maternal hypertension; AC, antenatal corticosteroids; AS, antenatal magnesium sulfate; PDA, patent ductus arteriosus; ROP, retinopathy of prematurity; RDS, respiratory distress syndrome; BPD, bronchopulmonary dysplasia; IVH, intraventricular hemorrhage; NEC, necrotizing enterocolitis; PVL, periventricular leukomalacia; WBC, white blood cell count; Hb, hemoglobin; PLT, platelet count.

### Fatty acid composition across gestational age groups

**Figure 2:**
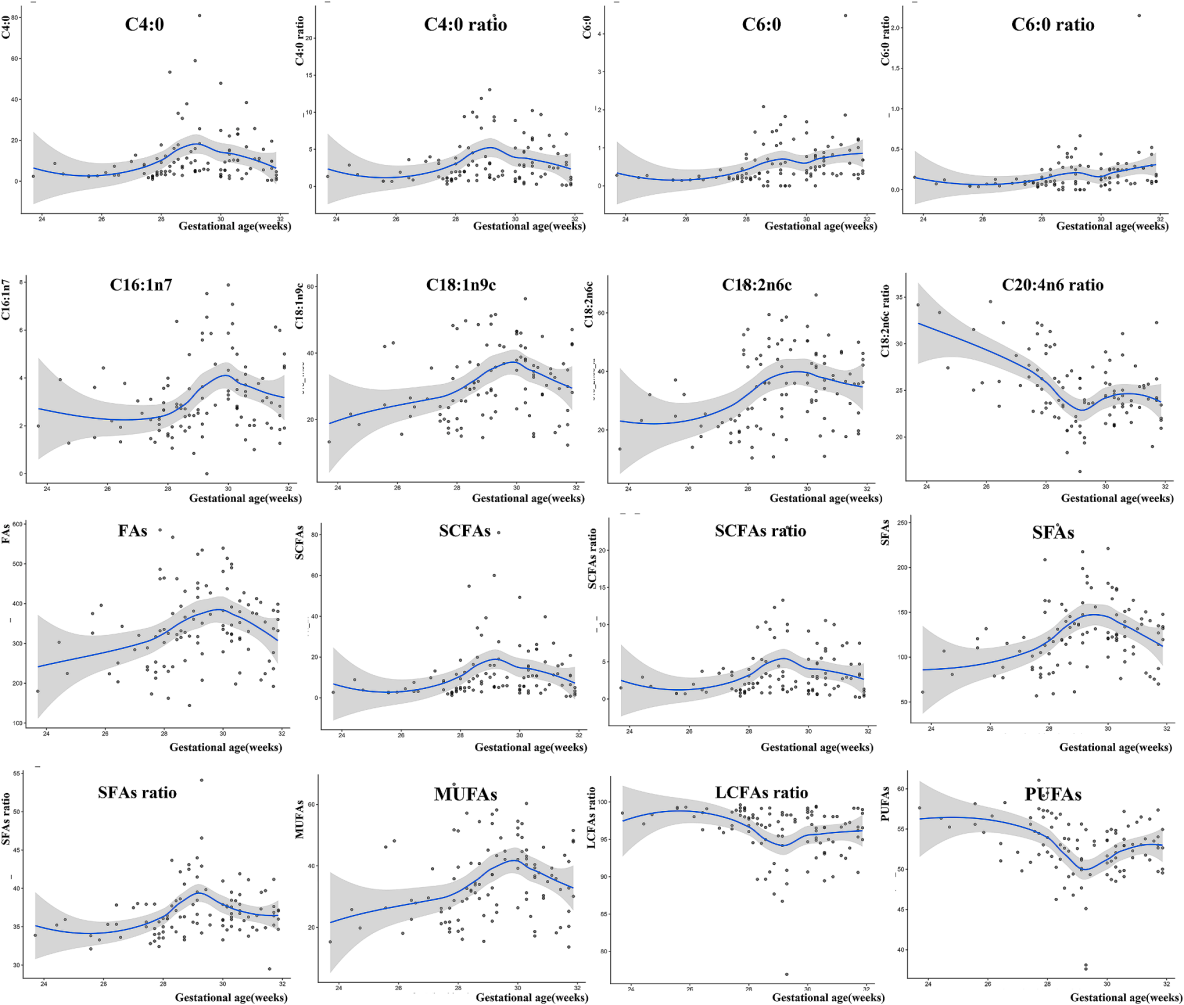
Fatty acid composition across gestational age groups C4:0, butyric acid; C6:0, caproic acid; C16:1n7, palmitoleic acid; C18:1n9c, oleic acid; C18:2n6c, linoleic acid; C20:4n6, arachidonic acid (ARA); FAs, fatty acids; SCFAs, short-chain fatty acids; SFAs, saturated fatty acids; MUFAs, monounsaturated fatty acids; LCFAs, long-chain fatty acids; PUFAs, polyunsaturated fatty acids.

Marked differences in fatty acid composition were observed across gestational age groups (Figure 2; Table S1). Total SCFAs increased with advancing gestational age (*p* = 0.003), with significant elevations in butyric acid (C4:0) and its relative proportion (both *p* = 0.004), as well as caproic acid (C6:0) and its proportion (*p* = 0.001 and 0.02). Total MCFAs and LCFAs did not differ significantly among groups; however, total SFAs increased with gestational age (*p* = 0.005). Within this class, palmitic acid (C16:0) peaked in the 28–30 weeks group (*p* = 0.06).

Total MUFAs increased with gestational age (*p* = 0.04), with significant elevations in oleic acid (C18:1n9c) and palmitoleic acid (C16:1n7) (*p* = 0.05 and 0.003). Although total PUFAs did not differ significantly (*p* = 0.4), linoleic acid (C18:2n6c) increased with gestational age (*p* = 0.01). Levels of ARA and DHA were comparable among groups; however, the proportion of ARA was significantly higher in the <28 weeks group (*p* < 0.001). Overall, total fatty acid concentrations tended to increase with gestational age, with a near-significant difference (*p* = 0.08).

### Perinatal factors influencing fatty acid composition in preterm infants

Several perinatal factors were found to influence the fatty acid profiles of preterm infants (Table 2; Tables S2 and S3). Maternal hypertension had a selective but significant impact on specific *n*-6 PUFAs, primarily reflected by an increased proportion of C18:2n6c (*p* = 0.001) and lower levels of eicosatrienoic acid (C20:3n6; *p* = 0.04) and ARA (*p* = 0.05). Infants of diabetic mothers exhibited an elevated ARA/DHA ratio (*p* = 0.003).

**Table 2:** Perinatal factors influencing fatty acid composition in preterm infants

	Estimate	Std.error	95% CI	*P*
**HIP**				

C18:2n6c ratio	1.82	0.55	0.73–2.91	0.001
C20:3n6	−2.58	1.25	−5.06–0.11	0.04
C20:4n6	−11.49	5.84	−23.08–0.10	0.05

**IDM**				

ARA/DHA ratio	0.24	0.11	0.02–0.46	0.03

**SEX**				

C20:3n6	−2.75	1.00	−4.73–0.77	0.009
C20:3n6ratio	−0.56	0.19	−0.94–0.19	0.003

**Multiple birth**				

C18:2n6c ratio	1.84	0.52	0.81–2.87	0.0007
C22:6n3	−7.08	2.85	−12.7–1.43	0.02
C22:6n3 ratio	−1.43	0.50	−2.42–0.43	0.005

**AC**				

C6:0	−0.40	0.16	−0.72–−0.08	0.02
C6:0ratio	−0.16	0.06	−0.30–0.04	0.02
C18:2n6c ratio	−1.99	0.64	−3.27–0.71	0.003
C20:4n6	15.3	6.8	1.64–28.97	0.03
C22:6n3	7.01	3.53	0.0003–14.02	0.05

**AS**				

C16:1n7	−0.67	0.32	−1.32–0.02	0.04
C16:1n7ratio	−0.16	0.08	−0.33–0.003	0.05

**Confounding adjustment:** HIP was adjusted for gestational age, IDM, AC, AS, sex, and multiple birth. IDM was adjusted for gestational age, HIP, AC, AS, sex, and multiple birth. SEX was adjusted for gestational age, HIP, IDM, AC, AS, and multiple birth. Multiple birth was adjusted for gestational age, HIP, IDM, AC, AS, and sex. AC was adjusted for gestational age, HIP, IDM, sex, AS, and multiple birth. AS was adjusted for gestational age, HIP, IDM, sex, AC, and multiple birth.

IDM, Maternal diabetes; HIP, Maternal hypertension; AC, Antenatal Corticosteroids; AS, Antenatal Magnesium Sulfate; C6:0, caproic acid; C16:1n7, palmitoleic acid; C18:2n6c, linoleic acid; C20:3n6, dihomo-gamma-linolenic acid (DGLA); C22:6n3, docosahexaenoic acid (DHA); ARA/DHA ratio, ratio of arachidonic acid to docosahexaenoic acid.

Multiple gestation demonstrated selective and directionally consistent alterations in fatty acid composition after adjustment for gestational age and other confounders, with a significantly higher proportion of C18:2n6c (*p* = 0.0007) and a markedly lower proportion of DHA (*p* = 0.005). With respect to sex differences, male preterm infants had lower absolute levels and proportions of eicosatrienoic acid (C20:3n6) than females (*p* = 0.009 and 0.003, respectively), while no significant sex-related differences were noted for other fatty acids.

Antenatal corticosteroid administration was associated with significant reductions in C6:0 and its proportion (both *p* = 0.02), as well as in the proportion of C18:2n6c (*p* = 0.003), and increased levels of ARA (*p* = 0.03) and DHA (*p* = 0.05). Exposure to antenatal magnesium sulfate was also associated with decreased levels and proportions of C16:1n7 (*p* = 0.04 and 0.05, respectively).

## Discussion

This study systematically characterized serum fatty acid profiles in preterm infants with a gestational age <32 weeks and examined their associations with perinatal factors. Our findings revealed significant differences in fatty acid composition among preterm infants of varying gestational ages, with levels of SCFAs, SFAs, and MUFAs increasing markedly with advancing gestation. Additionally, maternal comorbidities, antenatal medications, and infant sex influenced fatty acid profiles, indicating that both gestational age and perinatal environment play key roles in modulating fatty acid metabolism in preterm infants.

Although neonatal fatty acid metabolism has been a research focus, most existing studies have concentrated on the relationship between fatty acid profiles and adverse neonatal outcomes. In many of these studies, the measured fatty acid levels were largely influenced by exogenous interventions, such as lipid emulsions with different fatty acid compositions or enteral supplementation with long-chain fatty acids (e.g., DHA and ARA).^15^^,^^16^ Additionally, some studies have evaluated changes in breast milk fatty acid levels following such interventions,^17^ while others have focused on the LCFA profiles of preterm infants born to mothers with preeclampsia.^18^ A subset of studies has further examined the associations between maternal-fetal fatty acid status and adverse outcomes in preterm infants.^7^^,^^19^^,^^20^

Physiologically, an increase in fatty acid levels with advancing gestational age is expected. In our cohort, the trend approached significance. Limited attention has been given to the fatty acid status itself, particularly with respect to gestational age-dependent differences. Notably, one study comparing erythrocyte fatty acid composition between 30 preterm and 10 term infants found that preterm infants had significantly higher levels of SFAs and *n*-6 PUFAs, but lower levels of MUFAs and *n*-3 PUFAs at birth compared with term infants.^10^

Our study found that total long-chain and polyunsaturated fatty acids did not differ significantly across gestational age groups, whereas the proportion of linoleic acid increased with advancing gestational age. This pattern may be partly explained by the selective transport and metabolic regulation of fatty acids in the placenta. Transport-related proteins such as FATPs, FABPs, and LPL exhibit dynamic expression across gestation and may contribute to differential fatty acid distribution in the fetus.^21^ In addition, placental desaturases (e.g., FADS1 and FADS2) are involved in the conversion of linoleic acid into longer-chain *n*-6 PUFAs, and variations in their activity may influence the balance between linoleic acid and its downstream metabolites.^22^^,^^23^ These mechanisms may help maintain overall long-chain PUFA stability while allowing shifts in linoleic acid proportion.

Maternal supply of fatty acids to the fetus is tightly regulated to support rapid neural and tissue development during late gestation.^24^ Although the infants in this study were delivered prior to late gestation, they still exhibited a gestational age-dependent upward trend in fatty acid levels. Preterm birth interrupts this critical placental transfer, rendering extremely preterm infants particularly susceptible to multiple fatty acid deficiencies. Fatty acids such as DHA and ARA are essential for neurodevelopment, immune regulation, and intestinal integrity; their reduced levels in preterm infants have been closely associated with complications including impaired brain development, NEC, BPD, and ROP.^11^^,^^12^^,^^13^ Consequently, the lower fatty acid levels observed in extremely preterm infants are biologically plausible and consistent with evidence linking fatty acid imbalance to adverse neonatal outcomes. Nevertheless, the lower levels of SCFAs, SFAs, and MUFAs observed in infants of lower gestational age may reflect either pathological deficiency or a physiological characteristic of extreme prematurity. Further cohort studies focusing on clinical outcomes, together with comparative analyses including healthy term neonates as references for fatty acid levels, are warranted to clarify this distinction.

Preferential maternal-fetal transfer of LCFAs during late gestation is vital for optimal brain development. Maternal physiological conditions, including hypertension, diabetes, and multiple gestations, can alter lipid metabolism and thereby influence fatty acid transport and metabolism between mother and fetus.^25^^,^^26^ Our study found that maternal hypertension and diabetes were associated with alterations in fatty acid levels in preterm infants. However, detailed information on maternal dietary intake and early neonatal nutritional exposures, which may also affect infant serum fatty acid levels, was not available. In addition, the relatively limited sample size may have constrained the statistical power to detect weaker associations. Therefore, larger prospective studies with comprehensive assessments of maternal and neonatal nutrition and metabolic factors are warranted to further validate our findings.

A limited but significant sex-specific difference was observed for C20:3n6 after univariate analyses, including gestational age, maternal hypertension, maternal diabetes, antenatal corticosteroids, antenatal magnesium sulfate, and multiple births, suggesting potential sex-dependent regulation of fatty acid metabolism, possibly mediated by hormonal influences on fatty acid synthesis and catabolism.^27^ Extremely preterm infants generally display lower fatty acid levels and a heightened risk of adverse morbidities, as confirmed by our findings, emphasizing the critical importance of appropriate fatty acid supplementation. Given the widespread insufficiency of fatty acids in preterm infants—especially among extremely and very preterm populations—nutritional interventions should focus on the adequate and balanced provision of SCFAs, MCFAs, and LCFAs. Supplementation strategies should be individualized based on gestational age and further tailored according to maternal comorbidities and antenatal medication exposure. Compared with single fatty acid supplementation, interventions aimed at optimizing the overall fatty acid profile are likely to better support energy metabolism, immune modulation, and organ development, providing more comprehensive protection and developmental benefits for preterm infants.

Conclusively, in preterm infants with a gestational age below 32 weeks, serum fatty acid profiles exhibit a clear gestational age-dependent pattern. Maternal hypertension or diabetes, antenatal medications, and infant sex were significantly associated with specific fatty acid levels, indicating correlations between perinatal factors and fatty acid metabolism. These findings reveal gestational age-dependent differences in fatty acid profiles and the modulatory effects of maternal and perinatal factors, offering insights for future studies on fatty acid metabolism in preterm infants.

### Limitations of the study

Several limitations of this study should be acknowledged, and the findings interpreted with caution. First, the present results cannot determine whether the lower fatty acid levels observed in extremely preterm infants reflect pathological deficiency or a physiological characteristic of extreme prematurity. Further cohort studies examining clinical outcomes, as well as comparative analyses including healthy term neonates as references for fatty acid levels, are needed to clarify this issue. Second, detailed information on maternal dietary intake and early neonatal nutritional exposures, which may also influence infant fatty acid levels, was not available. The absence of these data as potential confounders may have limited the ability to fully evaluate the effects of maternal comorbidities and infant sex on fatty acid profiles. In addition, common metabolic-related factors such as maternal gestational obesity, hyperlipidemia, smoking, alcohol consumption, and blood glucose were not considered, and the dose-response relationship between antenatal medication use and fatty acid profiles could not be assessed. Thirdly, although the sample size is relatively large compared with similar studies, the single-center design may limit generalizability. Moreover, only early postnatal fatty acid levels were assessed, and the effects of long-term nutritional interventions on fatty acid profiles and clinical outcomes were not examined. Finally, measurements were confined to plasma fatty acids without the assessment of red blood cell membranes or tissue levels, potentially underestimating certain metabolic features. Future studies should adopt multicenter, large-scale longitudinal cohorts to dynamically monitor fatty acid profiles over time and investigate their associations with growth, development, and disease outcomes.

## Resource availability

### Lead contact

Further information and requests for resources and reagents should be directed to and will be fulfilled by the Lead Contact, DD (dangdan@jlu.edu.cn; Address: No.1 Xinmin Street, Changchun, Jilin Province, China; Telephone: +86–13894822116; ORCID: 0000-0001-5058-417X).

### Materials availability

This study did not generate new unique reagents.

### Data and code availability

• The authors declare that all data supporting the findings of this study are available within the article and its supplementary information files.
• This paper does not report original code.
• Other items: Any additional information required to reanalyze the data reported in this paper is available from the lead contact upon request.

## Acknowledgments

This research received funding from the 10.13039/501100011789Jilin Provincial Department of Science and Technology (YDZJ202301ZYTS070), the 10.13039/501100001809National Natural Science Foundation of China (82301952), and the Jilin Province Healthcare Talent Program (JLSRCZX2026-62).

## Author contributions

C.Z.: performed study concept and design, performed development of methodology, analysis and interpretation of data, writing – original draft, and read and approved the final paper.; Z.W. and H.W.: performed study concept and design, provided technical and material support, and read and approved the final paper.; L.X. and H.T.Y.: provided technical support and read and approved the final paper.; D.D.: performed study concept and design, project administration, writing – review and editing, and read and approved the final paper.

## Declaration of interests

The authors declare no competing interests.

## STAR★Methods

### Key resources table

**Table:** 

REAGENT or RESOURCE	SOURCE	IDENTIFIER
**Chemicals, peptides, and recombinant proteins**		

Methyl heptadecanoate	Sigma-Aldrich	51633
CH_3_OH	Sigma-Aldrich	1.06035
CH_3_NaO	Sigma-Aldrich	92446
C_6_H_14_	Sigma-Aldrich	1.04371
C_15_H_24_O	Sigma-Aldrich	B1378
CH_2_Cl_2_	Sigma-Aldrich	650463

**Software and algorithms**		

R software	version 4.5.1	https://www.r-project.org/

### Experimental model and study participant details

This prospective cohort study enrolled preterm infants with a gestational age <32 weeks who were admitted within 24 h of birth between September 2023 and September 2024. Infants with congenital metabolic disorders, major congenital malformations, or critical illness with an anticipated inability to survive were excluded. Informed consent was obtained from the parents or legal guardians of all participants. Within 24 h of life, routine hematological tests were performed, and residual blood samples were collected. Serum was separated by centrifugation and stored at −80°C for subsequent fatty acid analysis. The study protocol was approved by the Ethics Committee of the First Hospital of Jilin University (approval No. 23K153-001) and registered in the Clinical Trial Registry (registration No. NCT06045130).

### Method details

Baseline data were collected including both maternal pregnancy-related and neonatal characteristics. Maternal information consisted of age, pregnancy complications such as diabetes or hypertension, and multiple birth. Maternal diabetes or hypertension was diagnosed by obstetricians and documented in the medical records. Neonatal information included sex, gestational age, birth weight, and primary diagnoses. Mortality was defined as either in-hospital death or discharge against medical advice in the context of a clinical condition incompatible with survival, such as dependence on invasive or non-invasive ventilation, continuous vasoactive medication, or exclusive parenteral nutrition without initiation of enteral feeding. Intraventricular hemorrhage (IVH) was graded according to the Papile classification,^28^ while periventricular leukomalacia (PVL) was confirmed by neuroimaging (cranial ultrasound or MRI). BPD was defined in infants born at <32 weeks’ gestation as requiring cumulative oxygen supplementation by 36 weeks of corrected gestational age.^29^ Patent ductus arteriosus (PDA) was defined as a PDA requiring medical or surgical intervention. ROP was staged according to the third edition of the International Classification of Retinopathy of Prematurity,^30^ and necrotizing enterocolitis (NEC) was diagnosed using Bell’s criteria, stage II or III.^31^ PVL was assessed by radiologists, ROP by ophthalmologists, and the diagnosis and management of PDA as well as the diagnosis of NEC were determined by neonatologists, and all were documented in the medical records.

Fatty acid profiles were analyzed using gas chromatography-mass spectrometry (GC-MS; GCMS2010 Plus, Shimadzu). The detailed procedures for sample preparation and instrumental operation are described in a previous study.^32^ Separation was performed on a Supelco SP-2560 capillary column (Sigma), with methyl heptadecanoate (Sigma, 51633) used as the internal standard. Results were expressed as absolute concentrations and categorized according to carbon chain length or degree of unsaturation into short-chain fatty acids (SCFAs), medium-chain fatty acids (MCFAs), long-chain fatty acids (LCFAs), saturated fatty acids (SFAs), monounsaturated fatty acids (MUFAs), and polyunsaturated fatty acids (PUFAs).

### Quantification and statistical analysis

All statistical analyses were performed using R software (version 4.5.1; https://www.r-project.org/). The analyses utilized the following R packages: *readxl*, *dplyr*, *tableone*, *ggplot2*, *openxlsx*, and *broom*.

Sample size estimation was based on detecting a medium effect size (f = 0.25) among the three gestational-age groups. Under this assumption, approximately 96 infants would provide 80% power at a two-sided α level of 0.05.

Continuous variables with a normal distribution were expressed as mean ± standard deviation (SD), whereas non-normally distributed data were presented as median (interquartile range). Categorical variables were summarized as frequencies and percentages. Infants were stratified into three groups according to gestational age: <28 weeks, 28–30 weeks, and ≥30 weeks. Comparisons of baseline characteristics and fatty acid levels among gestational age groups were conducted using one-way analysis of variance for normally distributed continuous variables, the Kruskal–Wallis test for non-normally distributed data, and the χ^2^ test or Fisher’s exact test for categorical variables. Trend analyses of continuous variables were performed using trend χ^2^ tests or linear regression models.

To examine the influence of perinatal factors (e.g., maternal pregnancy complications, antenatal medication exposure, neonatal sex, and mode of delivery) on fatty acid profiles, multivariable linear regression models were constructed based on the results of the univariate analyses, with gestational age and several potential confounders included as covariates for adjustment. Results were reported as regression coefficients (β) with corresponding 95% confidence intervals (95% CI). All statistical tests were two-sided, and a *p*-value <0.05 was considered statistically significant.

### Additional resources

Clinical Trial Registry number: NCT06045130 (Study Details | NCT06045130 | PUFAs in Preterm Infants | ClinicalTrials.gov).

## Supplemental information

Table S1. Fatty acid composition across gestational age groups

Table S2. Baseline characteristics of the study population by group

Table S3. Perinatal factors influencing fatty acid composition in preterm infants
